# Supplementary material for: Post-translational covalent assembly of CAR and synNotch receptors for programmable antigen targeting
Source: Nat Commun. 2023 May 9;14:2463. doi: 10.1038/s41467-023-37863-5 (PMC10169838; doi:10.1038/s41467-023-37863-5)
Supplement: Supplementary file 1 — Supplementary Information [file 41467_2023_37863_MOESM1_ESM.pdf]

Supplementary Information for:

Post-translational covalent assembly of CAR and synNotch receptors for programmable antigen targeting

Elisa Ruffo, Adam A. Butchy, Yaniv Tivon, Victor So, Michael Kvorjak, Avani Parikh, Eric L. Adams, Natasa Miskov-Zivanov, Olivera J. Finn, Alexander Deiters, and Jason Lohmueller

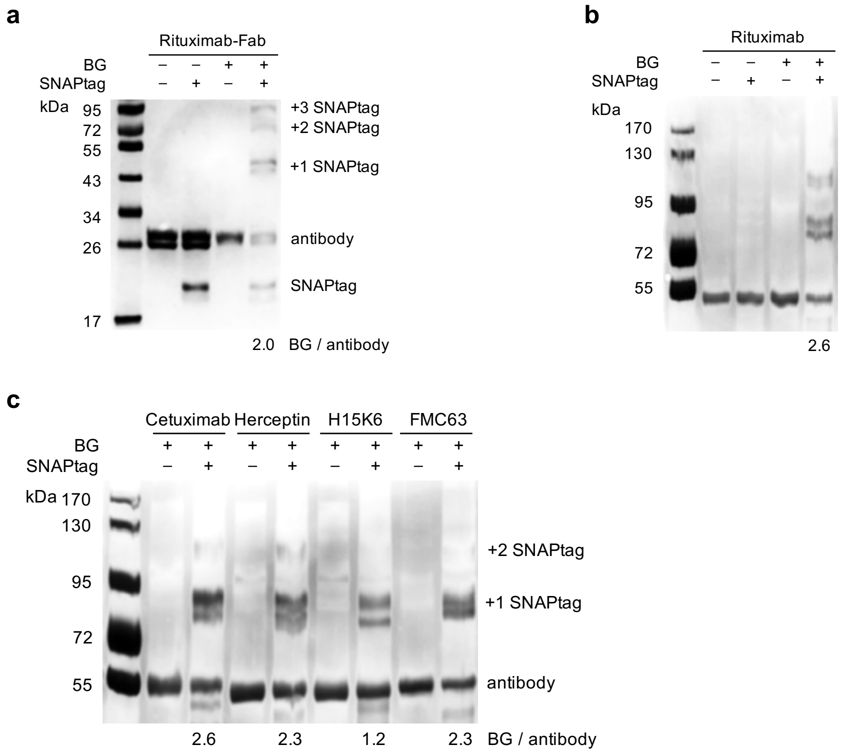

Figure S1 | SDS-PAGE quantification of antibody-conjugated benzyl guanines by SNAP conjugation reaction. Quantification of antibody benzylguanine (BG) labeling efficiency. SNAPtag conjugation to the light chain, **a**, or the whole antibody, **b**, was only achieved in the presence of both the BG-labeled antibody and the SNAPtag protein. A panel of BG-labeled antibodies, **c**, were then assessed in the same manner. Average number of BGs per antibody was calculated based on relative band intensities. The SDS-PAGE quantification gels are representative of a single experiment. Source data are available as a Source Data file.

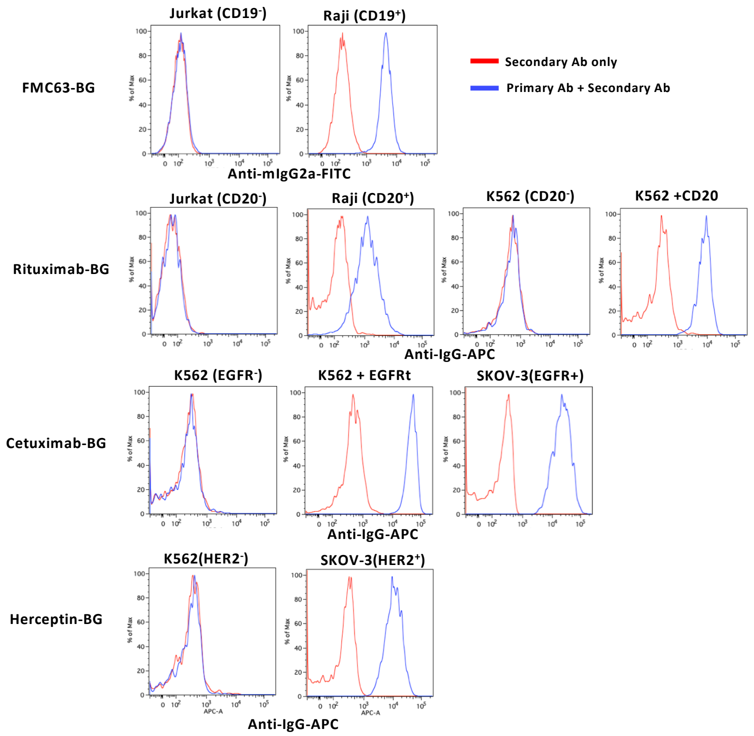

Figure S2 | Staining of target cell lines by BG-conjugated antibodies. Target cell lines were stained with 1.6 μg/mL of the indicated BG-conjugated antibodies (1.0 μg/mL was used for FMC63-BG) followed by staining with an anti-IgG secondary antibody (anti-mIgG2a-FITC for FMC63-BG and anti-IgG (Fab2)-APC for all other antibodies). Cells were then washed and analyzed by flow cytometry. Source data are available as a Source Data file.

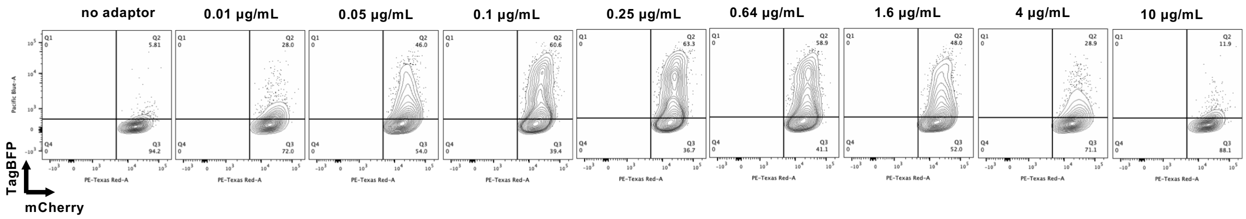

Figure S3 | Contour plots of FMC63-BG adaptor induction of TagBFP output by SNAP-synNotch cells. SNAP-synNotch cells were co-incubated with Raji cells and the indicated concentration of FMC63-BG adaptor for 48 hours. Cells were analyzed by flow cytometry and gated for mCherry expression. TagBFP output gene expression is plotted vs. mCherry (5% countour with outliers). Images correspond to a single replicate from the first panel of **Fig. 2d**. Source data are available as a Source Data file.

	SNAP-synNotch Cells (Millions)											
	1	0.5	0.1	0.01	1	0.5	0.1	0.01	1	0.5	0.1	0.01
Raji Cells (Millions)	0 μg/mL FMC63-BG				0.1μg/mL FMC63-BG				1.0μg/mL FMC63-BG			
1	431	463	551	541	748	955	1176	1518	1970	2877	4219	4434
0.5	485	525	557	505	979	1125	1939	1809	1813	2683	4567	5349
0.1	447	483	470	341	1158	1568	2537	3397	1218	1488	2264	2468
0.01	445	522	511	286	550	732	1016	1823	519	667	984	806

Color key (MFI)
6000
4500
3000
2000
1000
750
500
250
100

Figure S4 | Effector to target effect on SNAP-synNotch receptor activity. Flow cytometry analysis of the activation of SNAP-synNotch cells co-incubated with the indicated target cell lines and FMC63-BG antibody at the indicated cell numbers and antibody concentrations for 48 hours for TagBFP output gene expression reported as mean fluorescence intensity (MFI). Source data are available as a Source Data file.

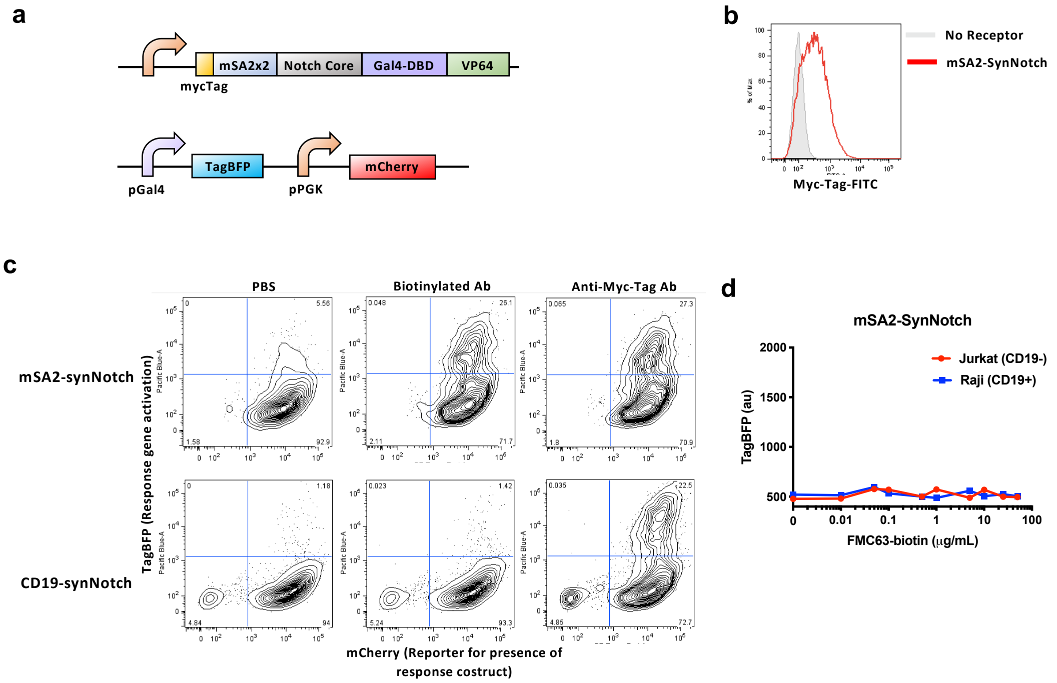

Figure S5 | mSA2 biotin-binding synNotch receptor is activated by plate-bound biotinylated antibody but not antibody bound to the surface of target cells. a, Design of SNAP-synNotch receptor expression and response lentiviral vectors. **b,** Flow cytometry analysis of the surface expression of the mSA2-synNotch receptor on transduced vs. MOCK (un)transduced Jurkat cells assessed by staining with the anti-Myc-Tag antibody. **c,** Flow cytometry analysis of the activation of mSA2-synNotch cells incubated on plates coated with PBS, biotinylated antibody, or anti-Myc-Tag antibody, for 48 hours for TagBFP output gene expression of response construct positive (mCherry+) cells. **d,** Flow cytometry analysis of the activation of mSA2-synNotch cells co-incubated with the indicated target cell lines and antibody concentrations for 48 hours for TagBFP output gene expression of response construct positive (mCherry+) cells reported as mean fluorescence intensity (MFI). Source data are available as a Source Data file.

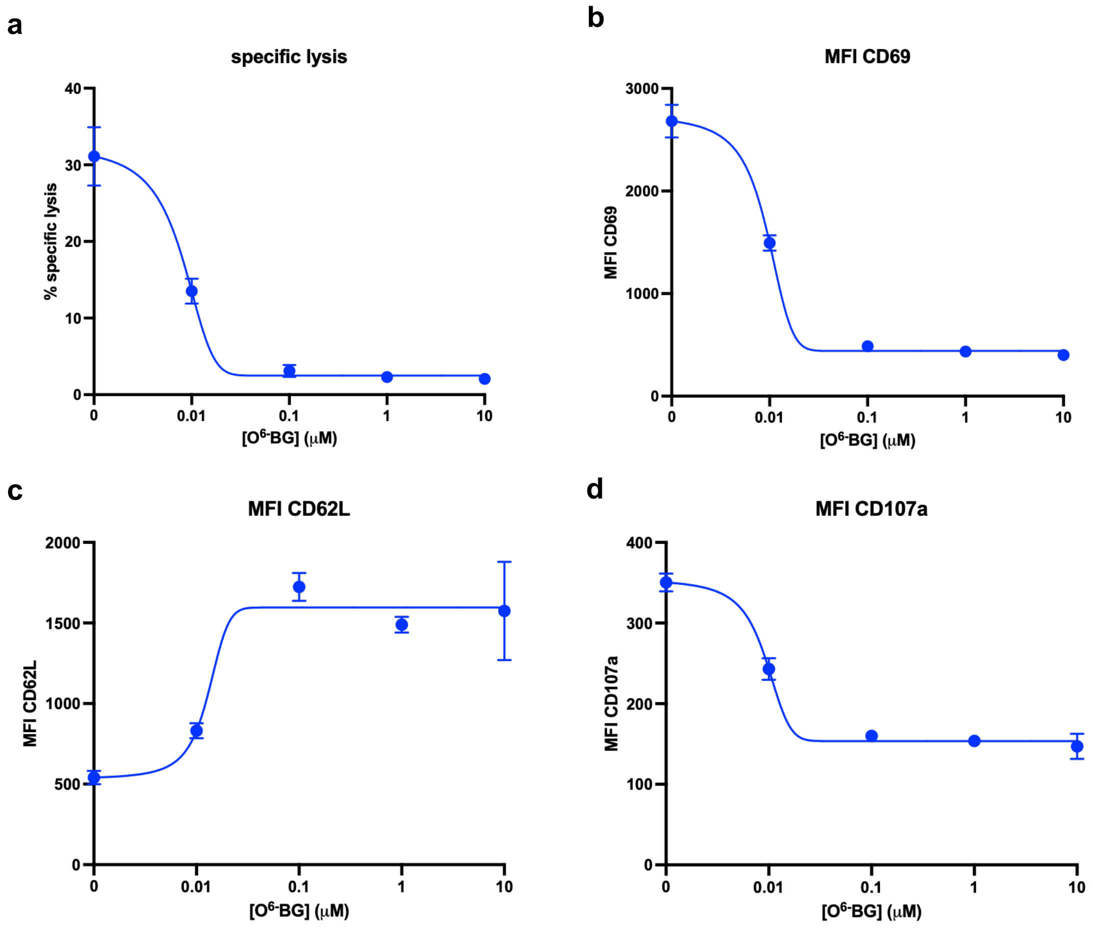

Figure S6 | O6-BG inhibits SNAP-CAR activity. a, Specific lysis of K562+HER2 cell line by co-incubating primary human SNAP-CAR T cells and 1μg/mL of Herceptin-BG antibody and indicated levels of O6-BG for 24 hours. **b**, **c**, **d** flow cytometry analysis of CD69, CD62L, and CD107a T cell activation markers on the SNAP-CAR T cells (LNGFR+ cell population) from co-incubation in **a**, reported as MFI, +/- std. deviation, n=3. Curve fits were performed in GraphPad Prism using non-linear regression "LogAbsoluteIC50" with base-line and top value constraints set by experimental values. Source data are available as a Source Data file.

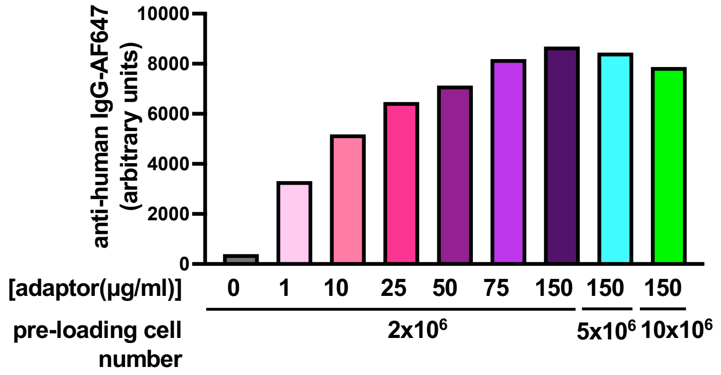

Figure S7 | Tuning SNAP-CAR T cell labeling by adaptor dose. Flow cytometry analysis of the indicated number of primary human SNAP-CAR CAR T cells co-incubated with varying doses of Rituximab-BG adaptor for 1 hour at 37°C, stained with a fluorescently labeled anti-human IgG-AF647 secondary antibody. Source data are available as a Source Data file.

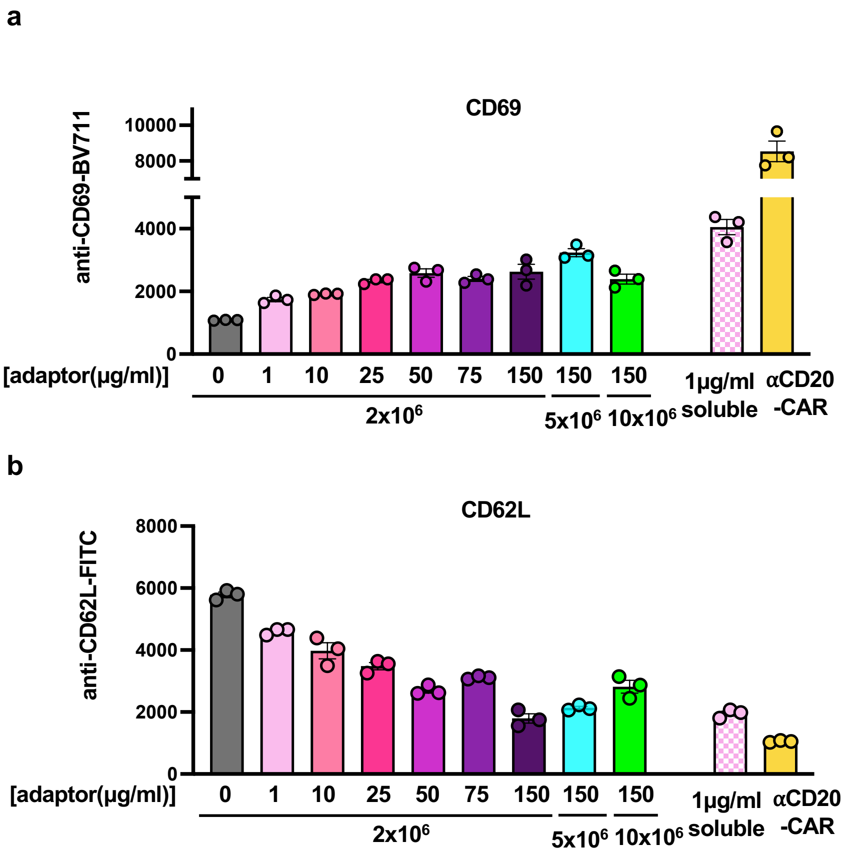

Figure S8 | Tuning T cell activation by adaptor dose. Flow cytometry analysis of primary human SNAP-CAR T cells at the indicated cell concentrations co-incubated with the indicated Rituximab-BG adaptor doses for 1hour at 37°C, followed by co-incubation with NALM6+CD20 target cells for 24 hours and staining for **a**, CD69, and **b**, CD62L markers on the SNAP-CAR T cells (LNGFR+ cell population), reported as MFI. n = 3 biologically-independent experiments ± s.e.m. Source data are available as a Source Data file.

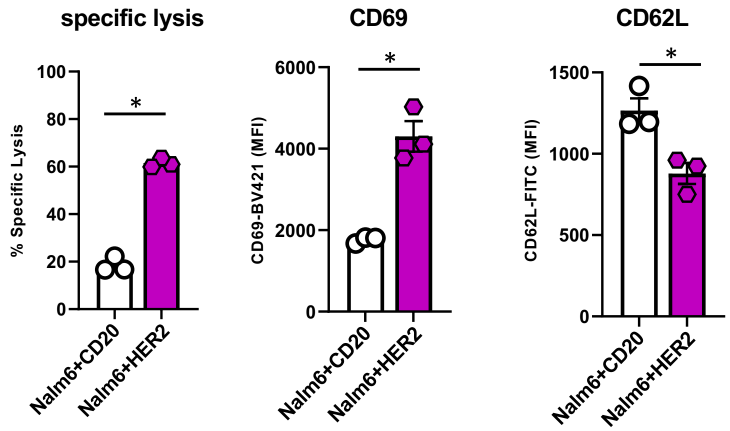

Figure S9 | Anti-HER2 CAR T cells show HER2-targeted effector function *in vitro*. Flow cytometry analysis of primary human anti-HER2 CAR T cells co-incubated with the indicated cell HER2+ or HER2(-) cell lines for 24 hours, for specific lysis of target cell lines and activation markers CD69, and CD62L markers on the SNAP-CAR T cell (LNGFR+ cell population), reported as MFI. n = 3 biologically-independent experiments ± s.e.m. To test significance, unpaired two-tailed student's t-tests were performed and " * " denotes a significance of $p < 0.05$. Exact p-values are $p<0.0001$ for specific lysis, $p=0.0025$ for CD69, and $p=0.0178$ for CD62L. Source data are available as a Source Data file.

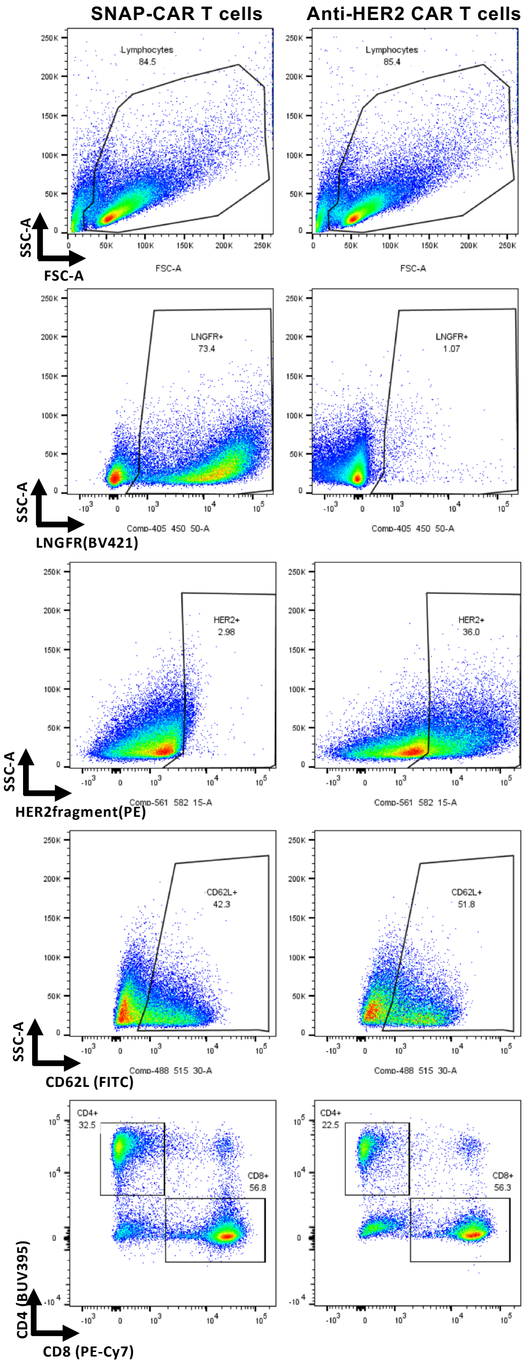

Figure S10 | CAR expression and T cell subset analysis of primary human CAR T cells. At day 5 post viral transduction (6 days prior to mouse injection), primary human CAR T cells were stained with the indicated antibodies and recombinant HER2-PE protein, washed, and analyzed by flow cytometry. Source data are available as a Source Data file.

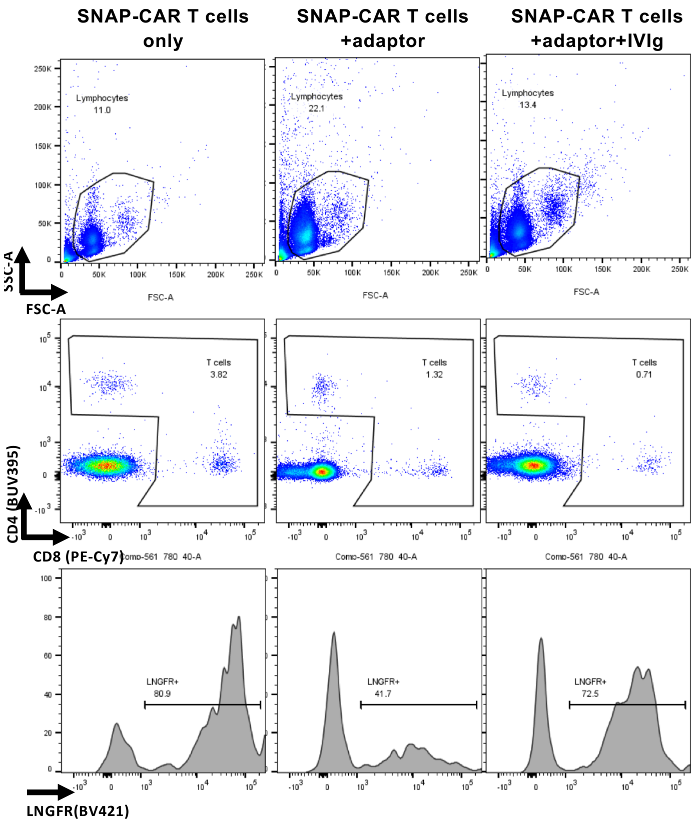

Figure S11 | IVIG co-injection maintains adoptively transferred SNAP-CAR T cells in the blood of mice. NSG mice were injected with SNAP-CAR T cells with or without Herceptin-BG antibody adaptor and IVIG. Following 24 hours mice underwent submandibular bleeding, RBC lysis, and washing. Isolated cells were stained with the anti-CD4, anti-CD8, and anti-LNGFR antibodies and analyzed via flow cytometry. Source data are available as a Source Data file.

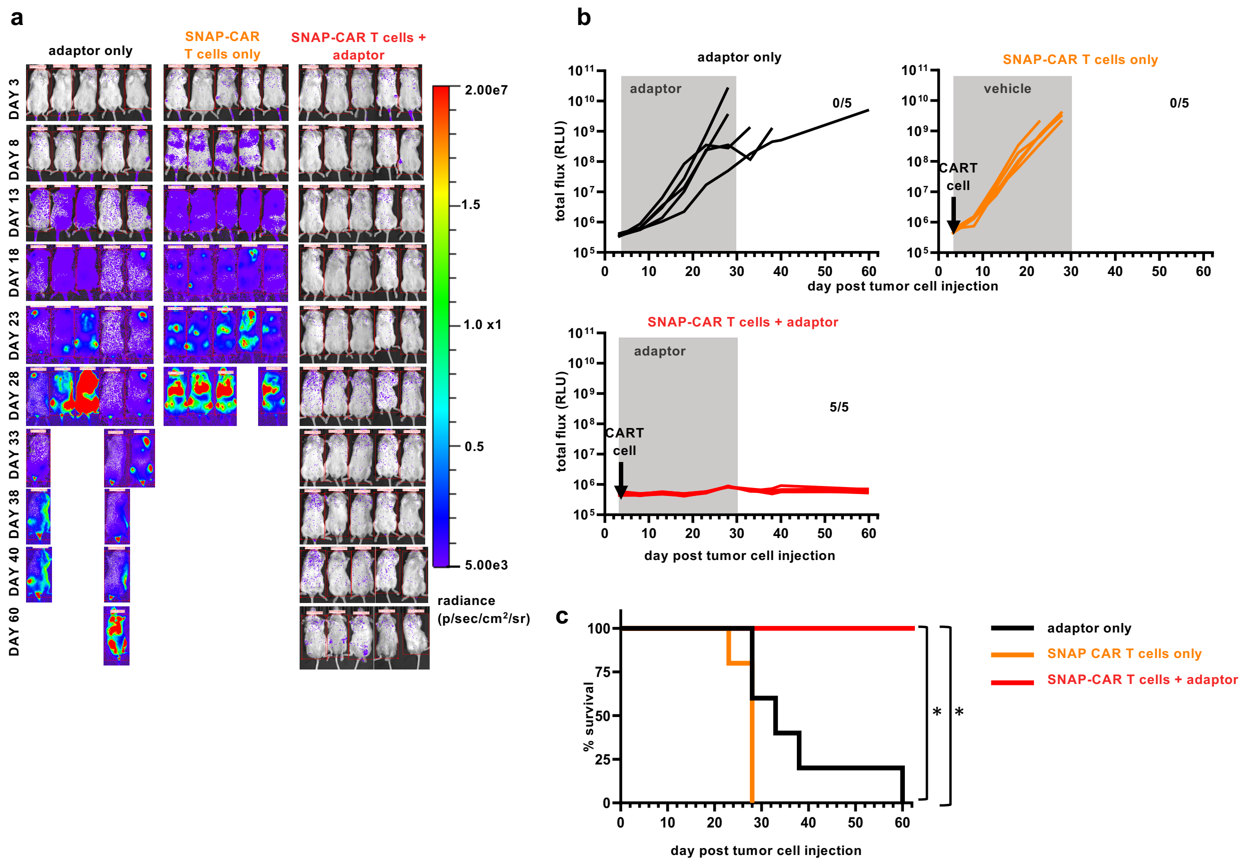

Figure S12 | Anti-tumor activity of SNAP-CAR T cells *in vivo* in a human tumor xenograft mouse model. a, IVIS imaging of tumor burden over time. **b,** Quantification of tumor growth via luciferase intensity (relative light units, RLU) for mouse images in **a**,. The number in the right side of the plot indicates the total number of mice that are tumor-free at day 60. **c**, Survival of treated mice over time. For **c,** a Mantel-Cox log-rank test was performed with a Bonferroni correction for multiple comparisons and " * " denotes a significance of $p < 0.025$ for 2 comparisons, $n = 5$ mice. Exact p-values are p=0.0017 for adaptor-only and p = 0.0035 for SNAP CAR T cells only. Source data are available as a Source Data file.

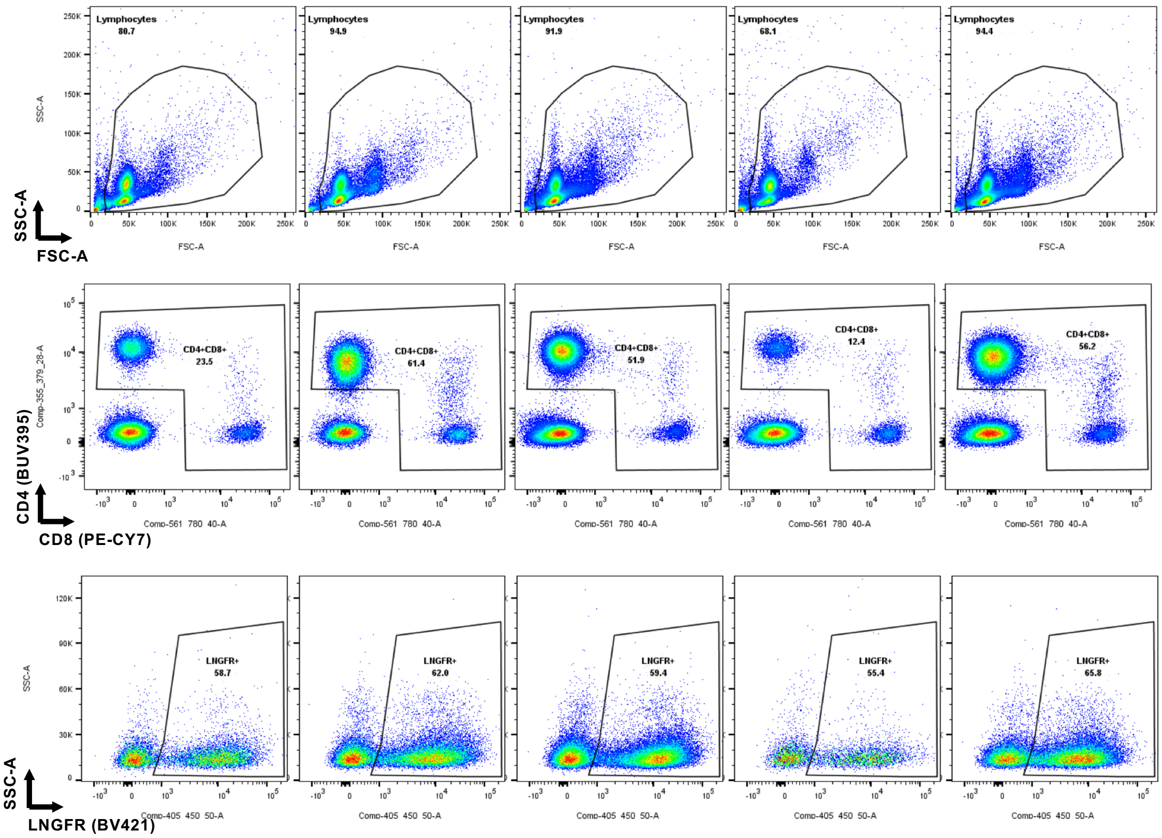

Figure S13 | Persistence of SNAP-CAR T cells *in vivo* in the blood of mice. Day 35 post tumor cell challenge, mice in the group treated with SNAP-CAR T cells + adaptor in Supplementary Fig. S12, underwent submandibular bleeding, RBC lysis, and washing. Isolated cells were stained with anti-CD4, anti-CD8, and anti-LNGFR antibodies and total human T cells (identified by CD4+ or CD8+) were analyzed via flow cytometry for % LNGFR-PE positive (CAR positivity). Each column corresponds to a single mouse. Source data are available as a Source Data file.

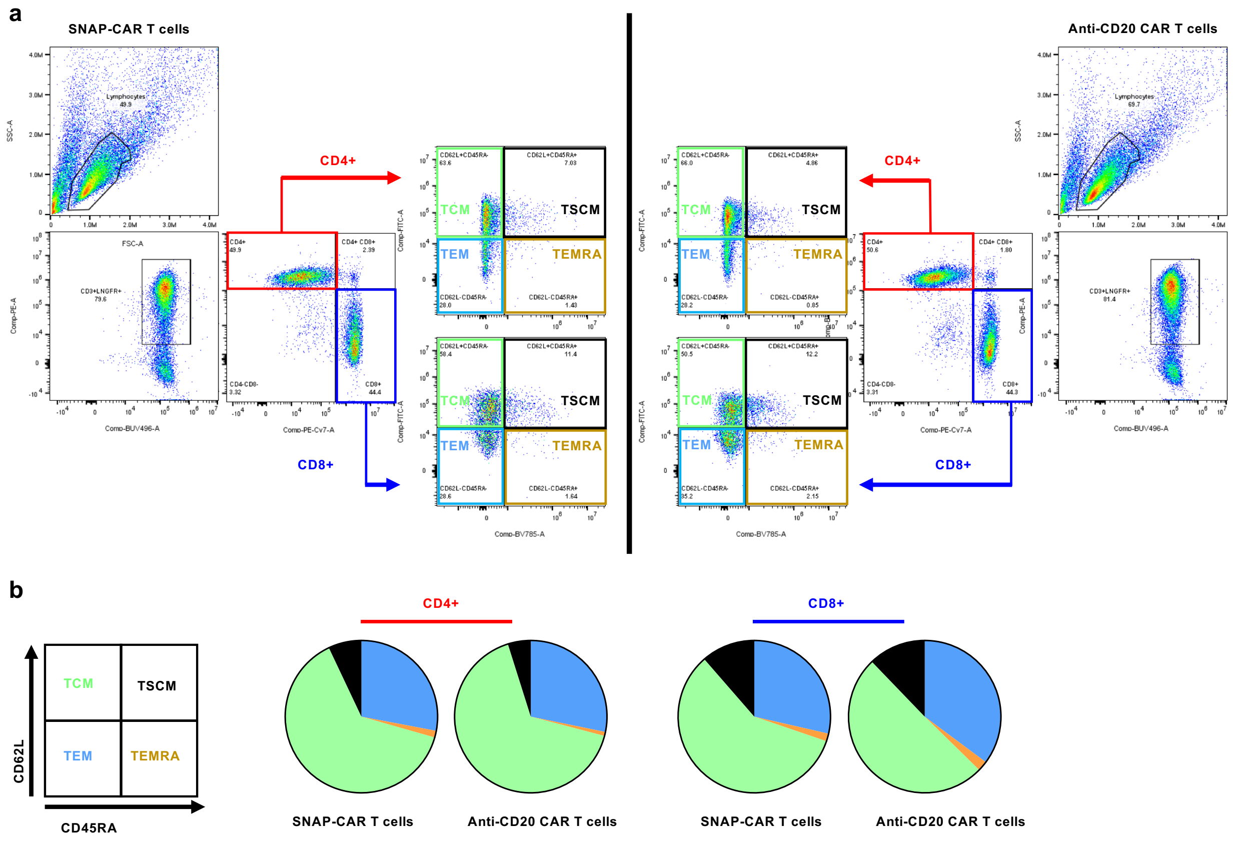

Figure S14 | T cell memory subsets of SNAP-CAR T cells compared to a traditional anti-CD20 CAR T cells. a, SNAP-CAR T cell and anti-CD20 CAR T cell phenotypes at day 7 post isolation. SNAP-CAR T cells and anti-CD20 CAR T cells were stained with anti-CD3, anti-CD4, anti-CD8, anti-LNGFR, anti-CD62L and anti-CD45RA antibodies and analyzed via flow cytometry. **b**, Gating scheme to identify putative T cell memory subsets, and pie charts for each cell population based on flow cytometry data in **a**. Source data are available as a Source Data file.

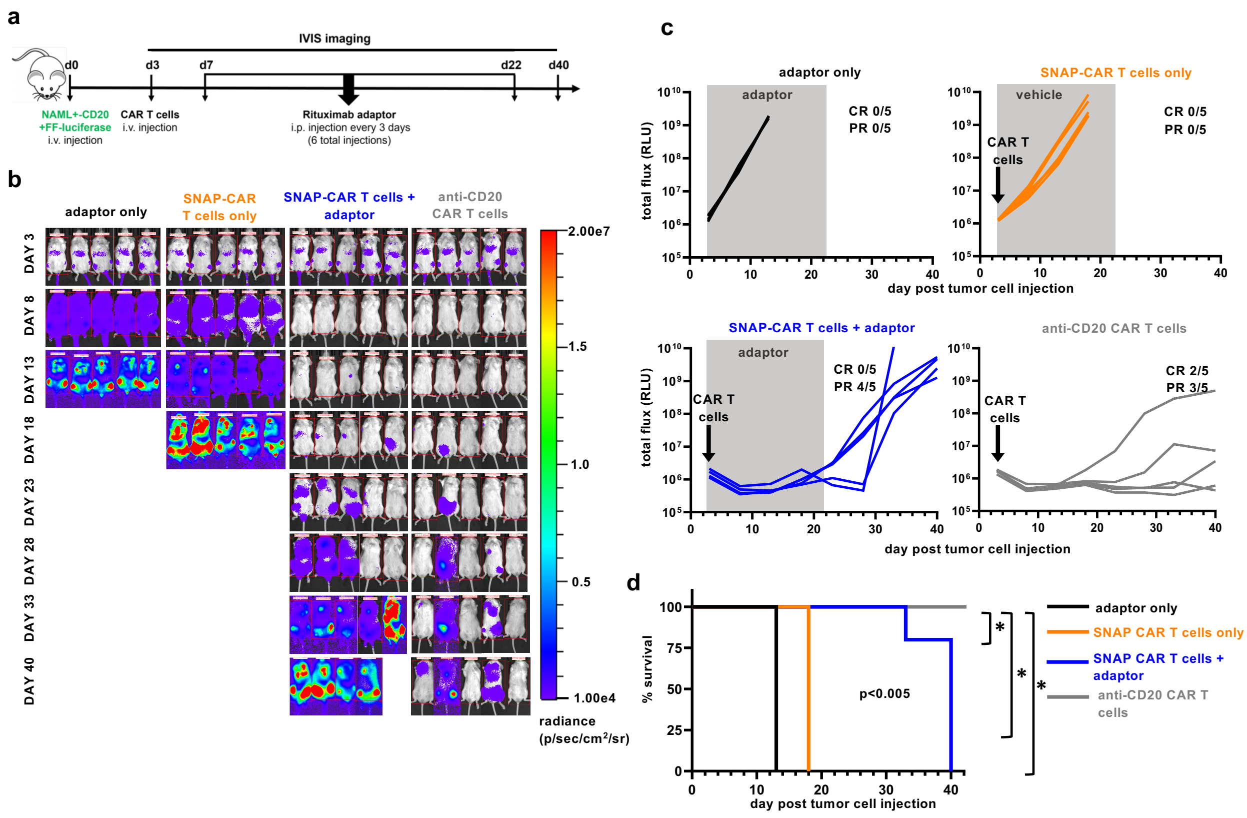

Figure S15 | Anti-tumor activity of SNAP-CAR T cells targeting CD20 *in vivo* in a human tumor xenograft mouse model. a, timeline and experimental design and **b**, IVIS imaging of tumor burden. **c**, Quantification of tumor growth via luciferase intensity (relative light units, RLU) for mouse images in **a**. PR” indicates partial response which is defined by a final tumor size over baseline but $<10^9$ at day 33. **d**, Survival of treated mice over time. **d**, a Mantel-Cox log-rank test was performed with a Bonferroni correction for multiple comparisons and “ * ” denotes a significance of $p < .01667$ for 3 comparisons, $n = 5$ mice per group. Exact p-values are p=0.0027 for adaptor-only, p = 0.0027 for SNAP CAR T cells only, and p = 0.0035 for anti-CD20 CAR T cells. Source data are available as a Source Data file.

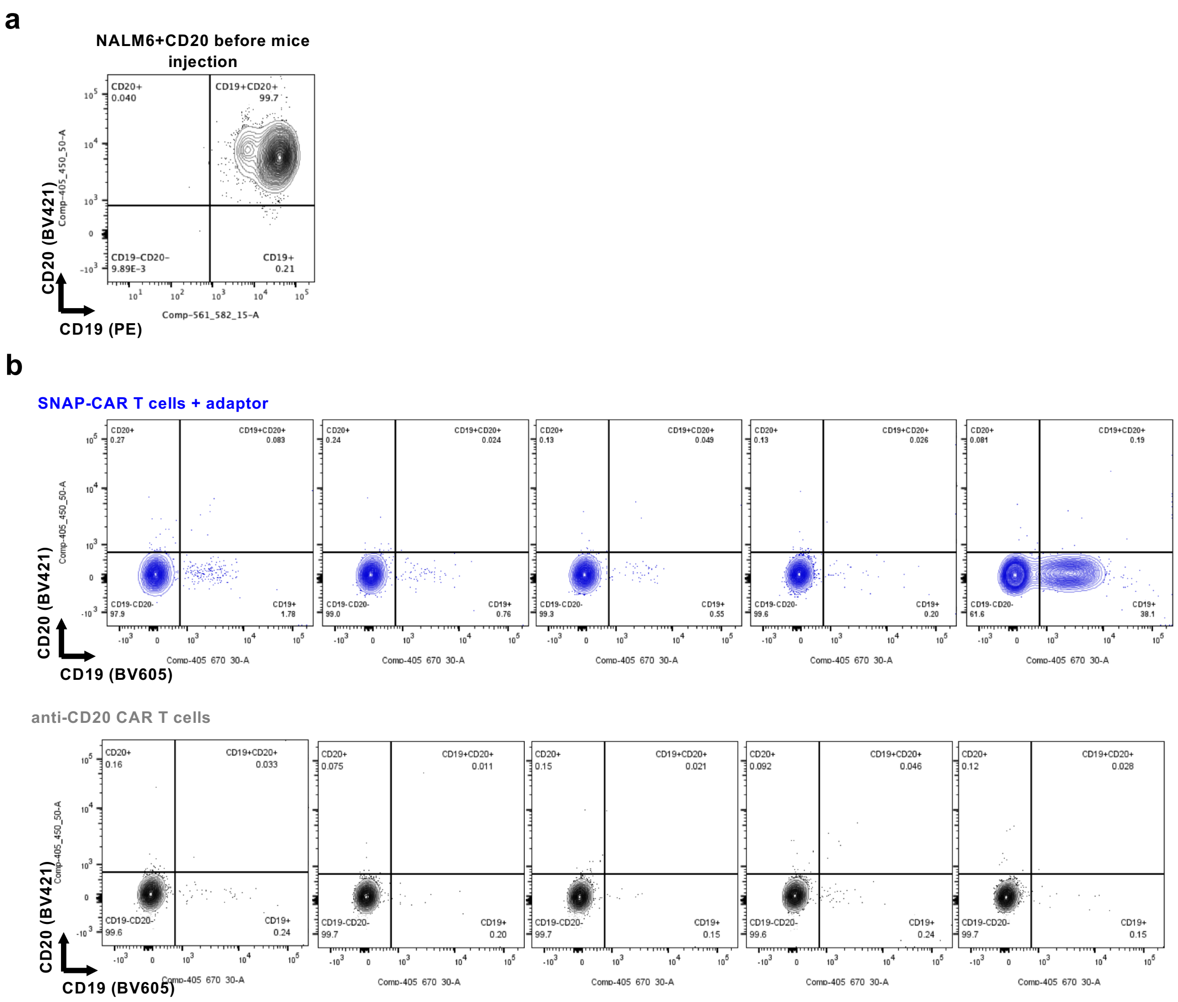

Figure S16 | Outgrowth of CD20(-) tumor cells in the blood of mice. a, Staining and flow cytometry analysis of NALM6+CD20 cells for CD19 and CD20 markers performed 1 day prior to their injection into mice. **b**, On day 40 post tumor cell injection, cells were harvested from the blood of mice from the indicated treatment groups. Isolated cells were then stained with the anti-CD19 and anti-CD20 antibodies and analyzed via flow cytometry. In **b**, each plot corresponds to a single mouse. Source data are available as a Source Data file.

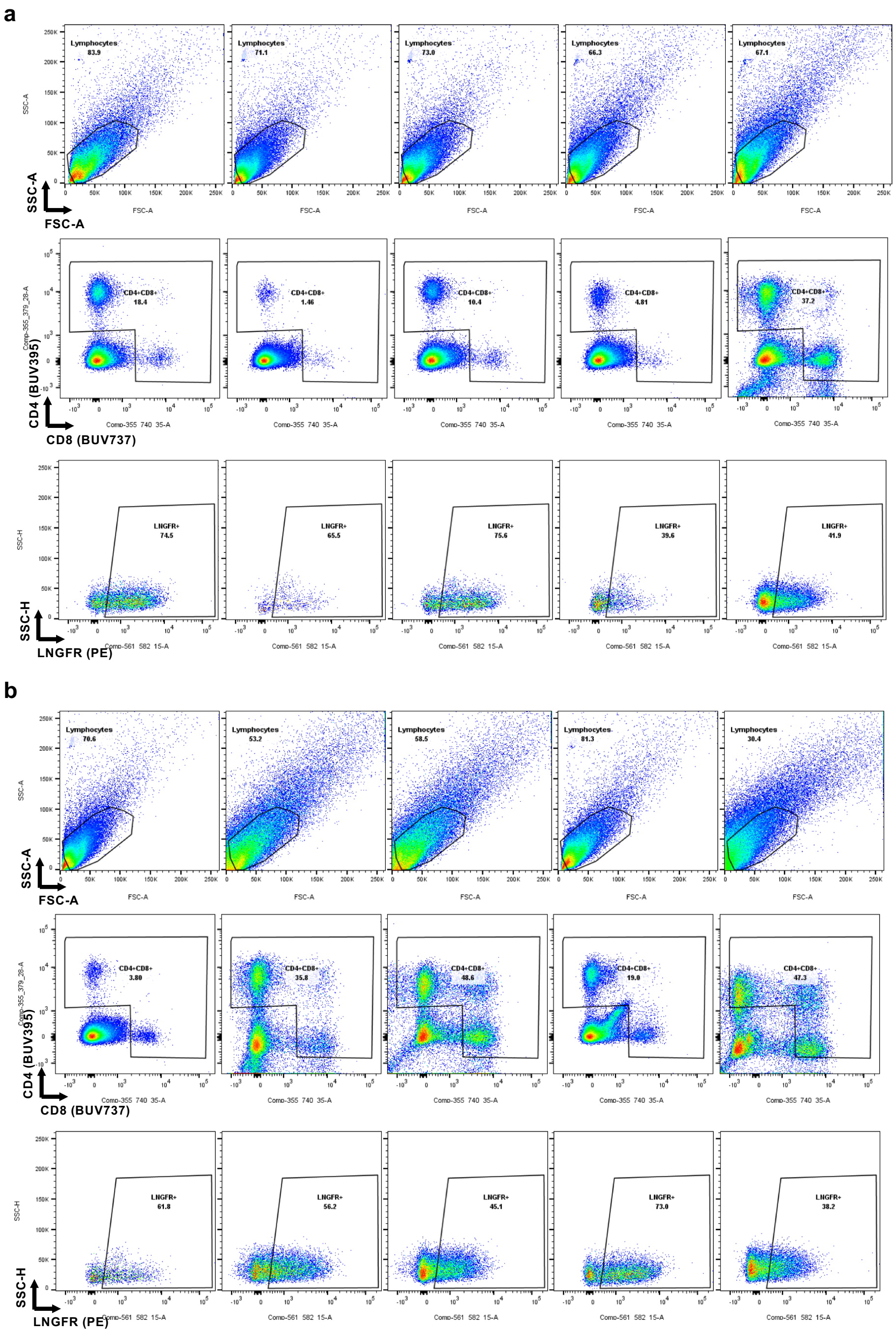

Figure S17 | Persistency of SNAP-CAR T cells *in vivo*. Day 40 post tumor cell challenge, splenocytes were harvested from groups of mice in Supplementary Fig. S15 treated with **a**, SNAP-CAR T cells + adaptor and **b**, CD20-CAR T cells. Isolated cells were stained with the anti-CD4, anti-CD8, and anti-LNGFR antibodies. Total human T cells (identified by CD4+ or CD8+) were

analyzed via flow cytometry for % LNGFR-PE positive (CAR positivity). Each column corresponds to a single mouse. Source data are available as a Source Data file.

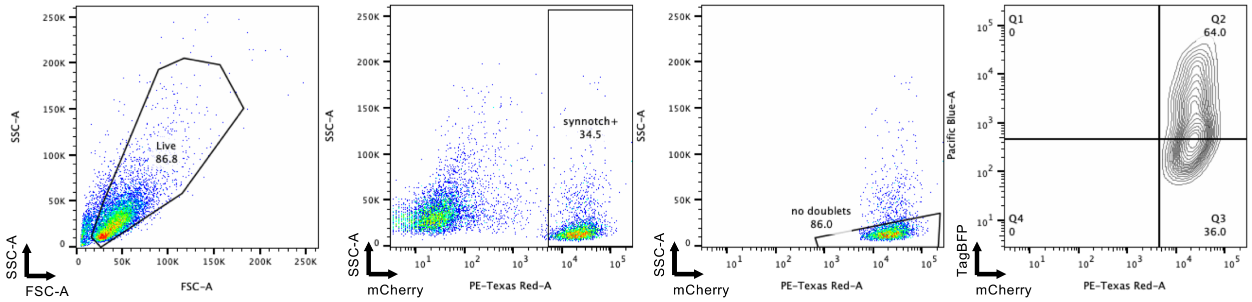

Figure S18 | Representative gating scheme for synNotch/target cell co-incubation assays.

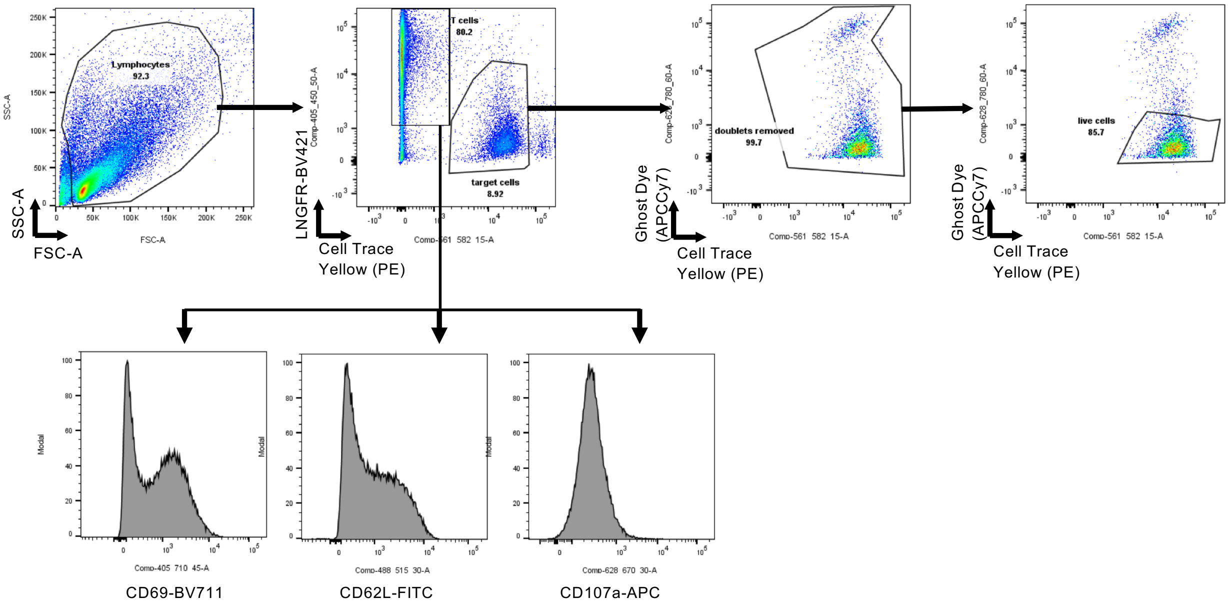

Figure S19 | Representative gating scheme for CAR T cell/target cell co-incubation assays.

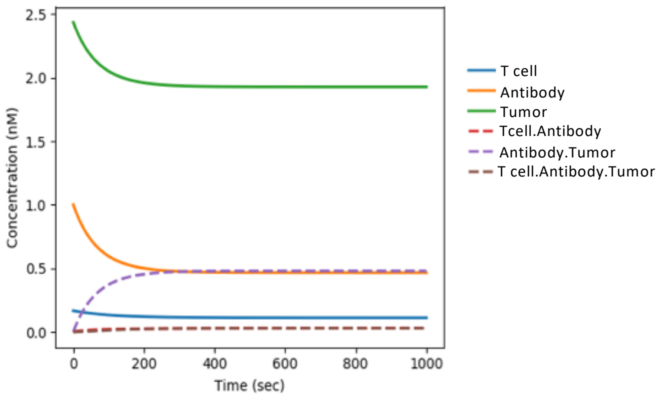

Figure S20 | Representative kinetic simulation. A kinetic simulation was performed using the experimental and literature values for the Cetuximab antibody and the EGFR antigen. All solid lines represent the species that are provided to the model while those in dotted lines represent complex species. Source data are available as a Source Data file.

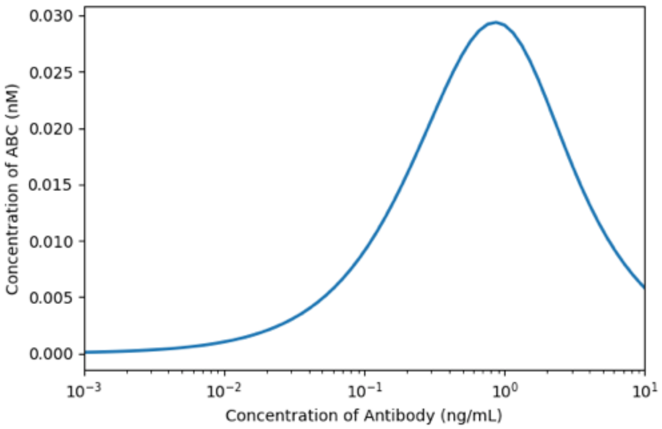

Figure S21 | Representative equilibrium simulation. An equilibrium simulation was performed using the experimental and literature values for the Cetuximab antibody and the EGFR antigen. "ABC" denotes the ternary body formation. Source data are available as a Source Data file.

Table S1 | Quantification of the number of BG molecules conjugated per antibody. Data summary from *Fig. S2.* Source data are available as a Source Data file.

Antibody	Antigen	# of BG/Antibody
Rituximab	CD20	2.8
FMC63	CD19	2
Cetuximab	EGFR	2.6
Herceptin	HER2	2.3
Rituximab-Fab	CD20	2.5

Table S2 | Model parameters used in simulations. Indicated parameters and their starting values as derived from the experimental set-up or from the literature. Source data are available as a Source Data file.

Experimental Setup		
Description	***Value***	***Units***
Well Volume	150	uL
Number of T Cells	150,000	cells
Number of Tumor Cells	400,000	cells
k_f for BG-site covalent bonding (Gautier 2008)	2.80 e-05	$nM^{-1} \cdot sec^{-1}$

Antigen Parameters	
Antigen	***Range***
CD19	10,000 – 500,000
CD20	100,000 – 300,000
EGFR	100,000 – 1,000,000
HER2	150,000 – 1,000,000

Kinetic Parameters					
Antibody	***Antigen***	k_f (nM^{-1} sec^{-1})	k_r (sec^{-1})	$K_{equilibrium}$ (nM)	***Citation***
Cetuximab	EGFR	3.10 e-03	5.80 e-03	1.87 e-00	Talavera 2009
FMC63	CD19	6.24 e-04	5.50 e-04	8.81 e-01	Kramer 2017
Herceptin	HER2	5.70 e-05	1.20 e-06	2.11 e-01	Bondza 2017
Rituximab	CD20	5.56 e-04	1.11 e-04	2.00 e-01	Melhus 2007

Table S3 | Model simulation error. Summary of sum of squared error (SSE) values calculated between the experimental results and simulations for simulations using parameters from the literature or parameters derived from model fitting. Source data are available as a Source Data file.

Model Error		
Simulation	***Literature Simulation***	***Fitted Simulation***
CAR with Cetuximab	0.38	0.38
CAR with FMC63	0.61	0.01
CAR with Herceptin	0.08	0.02
CAR with Rituximab	2.36	0.21
synNotch with Cetuximab	0.73	0.02
synNotch with FMC63	0.20	0.03
synNotch with Herceptin	1.39	0.02
synNotch with Rituximab	2.51	0.02

Table S4 | DNA sequences of gene constructs.

SNAP-41BBζ-T2A-TagBFP (entire coding region inserted into pHR-PGK vector)

**GGAGCAAGGCAGGTGGACAGTGGATCatggagacagacacactcctgctatgggtgctgctgctctgg
gttccaggttccacaggtATGGACAAAGATTGCGAGATGAAGAGAACCACCCTGGATAG
CCCTCTCGGCAAGCTCGAACTTTCTGGTTGTGAACAGGGTTTGCACAGGATC
ATCTTCCTGGGAAAGGGAACCTCAGCCGCAGATGCGGTTGAAGTGCCAGCTC
CGGCTGCAGTGCTTGGTGGACCCGAGCCTCTTATGCAAGCAACGGCATGGCT
TAATGCTTATTTTCACCAGCCTGAGGCCATTGAAGAGTTTCCAGTTCCTGCAT
TGCATCACCCCGTTTTTCAGCAGGAATCCTTCACTAGACAAGTGCTTTGGAA
GCTCTTGAAAGTGGTTAAATTTGGGGAAGTCATCTCATACAGCCACCTTGCT
GCCCTTGCAGGCAATCCTGCGGCCACGGCTGCAGTGAAAACTGCACTTAGCG
GAAATCCAGTCCCCATCTTGATACCGTGTCACAGGGTAGTACAGGGCGACCT
GGACGTCGGCGGTTACGAGGGCGGTTTGGCCGTTAAGGAATGGTTGCTGGC
GCATGAGGGTCACCGGCTGGGAAAACCAGGTCTTGGTGGAGGAAGTGGAGG
ATCTaccactactccggcaccgcgcccccaactcctgcaccgacgatagcttcacaaccgctttcattgcggcccgaagca
tgtcggccagccgccggaggcgctgtgcatacaagagggctggattttgcatgtgatatatatatttgggcgccccttgctggca
cttgcggcgttcttcttcttagcctcgttattacgctctactgtAAGCGAGGTAGGAAAAAATTGCTGTAT
ATCTTTAAACAGCCTTTTATGAGACCCGTGCAAACGACTCAAGAGGAAGACG
GGTGTAGCTGTAGATTTCCTGAAGAGGAAGAGGGGGGGTGCGAACTGcgggtga
agttcagcagaagcgccgacgcccctgcctaccagcagggccagaatcagctgtacaacgagctgaacctgggcagaaggg
aagagtacgacgtcctggataagcggagaggccgggaccctgagatgggcggcaagcctcggcggaagaacccccaggaa
ggcctgtataacgaactgcAgaaagacaagatggccgaggcctacagcgagatcggcatgaagggcgagcggaggcgggg
caagggccacgacggcctgtatcagggcctgtccaccgccaccaaggatacctacgacgccctgcacatgcaggccctgcccc
caaggCTCGAGGGCGGCGGAGAGGGCAGAGGAAGTCTTCTAACATGCGGTGAC
GTGGAGGAGAATCCCGGCCCTCGCatgagcgagctgattaaggagaacatgcacatgaagctgtacat
ggagggcaccgtggacaaccatcacttcaagtgcacatccgagggcgaaggcaagccctacgagggcacccagaccatgag
aatcaaggtggtcgagggcggccctctccccttcgccttcgacatcctggctactagcttcctctacggcagcaagaccttcatca
accacacccagggcatccccgacttcttcaagcagtccttccctgagggcttcacatgggagagagtcaccacatacgaagac
gggggcgtgctgaccgctacccaggacaccagcctccaggacggctgcctcatctacaacgtcaagatcagaggggtgaactt
cacatccaacggccctgtgatgcagaagaaaacactcggctgggaggccttcaccgagacgctgtaccccgctgacggcggc
ctggaaggcagaaacgacatggccctgaagctcgtgggcgggagccatctgatcgcaaacatcaagaccacatatagatcca
agaaacccgctaagaacctcaagatgcctggcgtctactatgtggactacagactggaaagaatcaaggaggccaacaacga
aacatacgtcgagcagcacgaggtggcagtggccagatactgcgacctccctagcaaactggggcacaagcttaattaaGA
TCCTTGACTTGCGGCCGCAACTCCCAC**

Legend:

5' region of pHR-PGK acceptor vector

Leader sequence and Kozak sequence

SNAPtag

CD8α-hinge,TM

4-1BBcyto

CD3ζcyto

T2A

TagBFP

3' region of pHR-PGK acceptor vector

pHR-PGK-SNAP-synNotch-Gal4-VP64 (insert for pHR–PGK-[]-synNotch-Gal4-VP64 backbone)

gagcaaaaacttatctctgaagaggacctcATGGACAAAGATTGCGAGATGAAGAGAACCACC
CTGGATAGCCCTCTCGGCAAGCTCGAACTTTCTGGTTGTGAACAGGGTTTGC
ACAGGATCATCTTCCTGGGAAAGGGAACCTCAGCCGCAGATGCGGTTGAAGT
GCCAGCTCCGGCTGCAGTGCTTGGTGGACCCGAGCCTCTTATGCAAGCAACG
GCATGGCTTAATGCTTATTTTCACCAGCCTGAGGCCATTGAAGAGTTTCCAGT
TCCTGCATTGCATCACCCCGTTTTTCAGCAGGAATCCTTCACTAGACAAGTGC
TTTGGAAGCTCTTGAAAGTGGTTAAATTTGGGGAAGTCATCTCATACAGCCA
CCTTGCTGCCCTTGCAGGCAATCCTGCGGCCACGGCTGCAGTGAAAACTGCA
CTTAGCGGAAATCCAGTCCCCATCTTGATACCGTGTCACAGGGTAGTACAGG
GCGACCTGGACGTCGGCGGTTACGAGGGCGGTTTGGCCGTTAAGGAATGGT
TGCTGGCGCATGAGGGTCACCGGCTGGGAAAACCAGGTCTTGGTGGAGGAA
GTGGAGGATCTatcctggactacagcttcacaggtggcgct

Legend:

5’ region of pHR_PGK_antiCD19_synNotch_Gal4VP64 acceptor vector

SNAPtag

3’ region of pHR_PGK_antiCD19_synNotch_Gal4VP64 acceptor vector

pHR-PGK-mSA2x2-synNotch-Gal4-VP64 (insert for pHR_PGK_[]_synNotch_ Gal4VP64 vector backbone)

gagcaaaaacttatctctgaagaggacctcGGCGCGGAGGCTGGAATCACTGGCACTTGGTAT
AATCAGCATGGCTCTACCTTTACTGTAACAGCAGGGGCTGATGGCAACCTTA
CCGGCCAATACGAGAATCGGGCGCAGGGTACGGGATGTCAAAATTCTCCCTA
TACACTGACCGGACGGTATAACGGTACCAAACTCGAATGGAGGGTAGAATGG
AACAACTCTACAGAAAACTGCCATAGCAGAACCGAATGGAGAGGCCAGTATC
AAGGAGGTGCAGAAGCCCGAATTAACACCCAATGGAACTTGACTTACGAAGG
CGGATCTGGACCAGCGACGGAACAGGGGCAGGATACTTTCACCAAAGTAAA
GCCAAGTGCTGCGTCCGGTAGTGAGGCCGCTGCTAAAGAAGCAGCGGCCAA
GGAAGCAGCTGCTAAGGGTGCAGAGGCTGGTATAACGGGGACTTGGTACAA
CCAGCACGGCTCCACCTTTACTGTGACTGCTGGGGCAGATGGGAACTTGACA
GGACAATATGAGAACCGAGCACAAGGGACGGGCTGCCAAAATAGTCCATATA
CACTCACGGGGCGATACAATGGCACTAAGCTGGAATGGAGGGTTGAGTGGA
ACAATTCAACGGAAAACTGTCATTCCCGCACTGAATGGCGGGGGCAGTACCA
GGGGGGGGCGGAGGCGAGAATCAACACACAATGGAACTTGACATACGAGGG
GGGAAGTGGGCCTGCCACCGAACAAGGACAGGACACTTTTACTAAAGTGAAG
CCCTCAGCTGCGTCAGGGAGTatcctggactacagcttcacaggtggcgct

Legend:

5’ region of pHR_PGK_antiCD19_synNotch_Gal4VP64 acceptor vector

mSA2x2

3’ region of pHR_PGK_antiCD19_synNotch_Gal4VP64 acceptor vector

pHR-pGal-IL7-PGK-mCherry (insert for pHR_Gal4UAS_[]_PGK_mCherry vector backbone)

**CCGATCCAGCCTCTCGACATTCGTTGGATCatgttccacgtaagtttcagatatatctttggacttcc
gccgctcatattggtattgttgccagtggcatctagtgactgtgacatagaaggaaaggatggtaaacagtatgaaagcgtactt
atggtatccattgaccagcttctcgatagtatgaaagagattggtagtaattgcctcaataacgagttcaatttctttaaacgaca
catttgtgatgcgaataaagagggaatgtttctgtttcgcgccgcgaggaagcttaggcagttccttaaaatgaactcaactggg
gatttcgacctccatctgctgaaggtgagtgaaggtactactattctcctgaattgcacgggacaggtaaaggggcgaaacct
gcggccttgggtgaggcacaaccaaccaaaagcctcgaagaaaacaagtccctcaaagaacagaagaagctcaacgatctg
tgctttctgaaaagactcttgcaggagatcaaaacttgttggaataagattttgatgggcactaaggagcatTAAGATCC
TTGACTTGCGGCCGCAACTCCC**

<u>SNAP-41BBζ-T2A-LNGFR (entire coding region inserted into MSGV1 vector, AgeI and NotI sites)</u>

**ACCGGTGGTACCTCACCCTTACCGAGTCGGCGACACAGTGTGGGTCCGCCGA
CACCAGACTAAGAACCTAGAACCTCGCTGGAAAGGACCTTACACAGTCCTGC
TGACCACCCCCACCGCCCTCAAAGTAGACGGCATCGCAGCTTGGATACACGC
CGCCCACGTGAAGGCTGCCGACCCCACGCGTCAAGTGGAGCAAGGCAGGTG
GACAGTGGATCatggagacagacacactcctgctatgggtgctgctgctctgggttccaggttccacaggtATGG
ACAAAGATTGCGAGATGAAGAGAACCACCCTGGATAGCCCTCTCGGCAAGCT
CGAACTTTCTGGTTGTGAACAGGGTTTGCACAGGATCATCTTCCTGGGAAAG
GGAACCTCAGCCGCAGATGCGGTTGAAGTGCCAGCTCCGGCTGCAGTGCTTG
GTGGACCCGAGCCTCTTATGCAAGCAACGGCATGGCTTAATGCTTATTTTCA
CCAGCCTGAGGCCATTGAAGAGTTTCCAGTTCCTGCATTGCATCACCCCGTT
TTTCAGCAGGAATCCTTCACTAGACAAGTGCTTTGGAAGCTCTTGAAAGTGG
TTAAATTTGGGGAAGTCATCTCATACAGCCACCTTGCTGCCCTTGCAGGCAA
TCCTGCGGCCACGGCTGCAGTGAAAACTGCACTTAGCGGAAATCCAGTCCCC
ATCTTGATACCGTGTCACAGGGTAGTACAGGGCGACCTGGACGTCGGCGGTT
ACGAGGGCGGTTTGGCCGTTAAGGAATGGTTGCTGGCGCATGAGGGTCACC
GGCTGGGAAAACCAGGTCTTGGTGGAGGAAGTGGAGGATCTaccactactccggcacc
gcgcccccaactcctgcaccgacgatagcttcacaaccgctttcattgcggcccgaagcatgtcggccagccgccggaggcg
ctgtgcatacaagagggctggattttgcatgtgatatatatatttgggcgccccttgctggcacttgcggcgttcttcttcttagcct
cgttattacgctctactgtAAGCGAGGTAGGAAAAAATTGCTGTATATCTTTAAACAGCC
TTTTATGAGACCCGTGCAAACGACTCAAGAGGAAGACGGGTGTAGCTGTAGA
TTTCCTGAAGAGGAAGAGGGGGGGTGCGAACTGcgggtgaagttcagcagaagcgccgacgc
ccctgcctaccagcagggccagaatcagctgtacaacgagctgaacctgggcagaagggaagagtacgacgtcctggataag
cggagaggccgggaccctgagatgggcggcaagcctcggcggaagaacccccaggaaggcctgtataacgaactgcAgaa
agacaagatggccgaggcctacagcgagatcggcatgaagggcgagcggaggcggggcaagggccacgacggcctgtatc
agggcctgtccaccgccaccaaggatacctacgacgccctgcacatgcaggccctgcccccaaggCTCGAGGGCGG
CGGAGAGGGCAGAGGAAGTCTTCTAACATGCGGTGACGTGGAGGAGAATCC
CGGCCCTCGCatgggtgcaggcgctacaggtcgcgcaatggatggccctagactgctccttcttctgttgctcggggttt
ccttgggtggcgcaaaggaagcatgccctacagggctgtacacccattccggcgaatgttgcaaagcgtgtaacctgggagag
ggtgttgctcaaccttgtggggccaaccagaccgtttgtgagccatgcctggatagcgttaccttcagcgacgtggtgtccgcca
ccgagccttgtaaaccctgcactgaatgcgtaggattgcaatctatgtccgccccctgcgttgaggcagacgacgccgtttgcag
gtgcgcctacggttactatcaggatgagacaaccggcagatgcgaagcatgtcgagtttgcgaggccggatcaggtctggtttt
cagctgtcaggataaacaaaataccgtatgtgaggaatgcccagacggaacctactccgatgaagccaaccacgtcgatccgt
gcctgccgtgcaccgtttgcgaggacacagaaaggcagctgcgagagtgcactcggtgggccgacgccgaatgcgaagaaat
ccctggacgctggatcaccaggtctactccccctgaagggagtgactcaactgcaccttcaacccaggagcctgaagctccccc
agagcaggacctcatcgcttcaacagtggccggggtggtcactacggtgatggggagttctcagcctgtggtcacacgaggca
ccacagataacctcattccggtttactgctcaatcttggcggcagttgtggttggtctggtggcgtatatcgcgtttaaaagatgga**

atagctagtagAAGTCGGGATCCTTGACTTGCGGCCGC

Legend:
5' region
Leader sequence
SNAPtag
CD8α-hinge,TM
4-1BBcyto
CD3ζcyto
T2A
LNGFR
3' region

Anti-CD20-41BBζ-T2A-LNGFR (entire coding region inserted into MSGV1 vector, AgeI and NotI sites)

ACCGGTGGTACCTCACCCTTACCGAGTCGGCGACACAGTGTGGGTCCGCCGA
CACCAGACTAAGAACCTAGAACCTCGCTGGAAAGGACCTTACACAGTCCTGC
TGACCACCCCCACCGCCCTCAAAGTAGACGGCATCGCAGCTTGGATACACGC
CGCCCACGTGAAGGCTGCCGACCCCACGCGTCAAGTGGAGCAAGGCAGGTG
GACAGTGGATCatggagacagacacactcctgctatgggtgctgctgctctgggttccaggttccacaggtGGTG
ACATTGTGCTGACCCAATCTCCAGCTATCCTGTCTGCATCTCCAGGGGAGAA
GGTCACAATGACTTGCAGGGCCAGCTCAAGTGTAAATTACATGGACTGGTAC
CAGAAGAAGCCAGGATCCTCCCCCAAACCCTGGATTTATGCCACATCCAACC
TGGCTTCTGGAGTCCCTGCTCGCTTCAGTGGCAGTGGGTCTGGGACCTCTTA
CTCTCTCACAATCAGCAGAGTGGAGGCTGAAGATGCTGCCACTTATTACTGC
CAGCAGTGGAGTTTTAATCCACCCACGTTCGGAGGGGGGACCAAGCTGGAAA
TAAAAGGCAGTACTAGCGGTGGTGGCTCCGGGGGCGGTTCCGGTGGGGGCG
GCAGCAGCGAGGTGCAGCTGCAGCAGTCTGGGGCTGAGCTGGTGAAGCCTG
GGGCCTCAGTGAAGATGTCCTGCAAGGCTTCTGGCTACACATTTACCAGTTA
CAATATGCACTGGGTAAAGCAGACACCTGGACAGGGCCTGGAATGGATTGGA
GCTATTTATCCAGGAAATGGTGATACTTCCTACAATCAGAAGTTCAAAGGCA
AGGCCACATTGACTGCAGACAAATCCTCCAGCACAGCCTACATGCAGCTCAG
CAGCCTGACATCTGAGGACTCTGCGGACTATTACTGTGCAAGATCTAATTATT
ACGGTAGTAGCTACTGGTTCTTCGATGTCTGGGGCGCAGGGACCACGGTCAC
CGTCTCCTCAaccactactccggcaccgcgcccccaactcctgcaccgacgatagcttcacaaccgctttcattgcgg
cccgaagcatgtcggccagccgccggaggcgctgtgcatacaagagggctggattttgcatgtgatatatatatttgggcgccc
cttgctggcacttgcggcgttcttcttcttagcctcgttattacgctctactgtaagcgaggtaggaaaaattgctgtatatcttta
aacagccttttatgagacccgtgcaaacgactcaagaggaagacgggtgtagctgtagatttcctgaagaggaagaggggg
gtgcgaactgcgggtgaagttcagcagaagcgccgacgcccctgcctaccagcagggccagaatcagctgtacaacgagctg
aacctgggcagaagggaagagtacgacgtcctggataagcggagaggccgggaccctgagatgggcggcaagcctcggcg
gaagaacccccaggaaggcctgtataacgaactgcAgaaagacaagatggccgaggcctacagcgagatcggcatgaagg
gcgagcggaggcggggcaagggccacgacggcctgtatcagggcctgtccaccgccaccaaggatacctacgacgccctgc
acatgcaggccctgcccccaaggctcgagggcggtggagagggccggggttctctcttgacctgtggggatgtagaagaaaac
cccgggccccgaATGGGTGCAGGCGCTACAGGTCGCGCAATGGATGGCCCTAGACT
GCTCCTTCTTCTGTTGCTCGGGGTTTCCTTGGGTGGCGCAAAGGAAGCATGC
CCTACAGGGCTGTACACCCATTCCGGCGAATGTTGCAAAGCGTGTAACCTGG
GAGAGGGTGTTGCTCAACCTTGTGGGGCCAACCAGACCGTTTGTGAGCCATG

CCTGGATAGCGTTACCTTCAGCGACGTGGTGTCCGCCACCGAGCCTTGTAAA
CCCTGCACTGAATGCGTAGGATTGCAATCTATGTCCGCCCCCTGCGTTGAGG
CAGACGACGCCGTTTGCAGGTGCGCCTACGGTTACTATCAGGATGAGACAAC
CGGCAGATGCGAAGCATGTCGAGTTTGCGAGGCCGGATCAGGTCTGGTTTTC
AGCTGTCAGGATAAACAAAATACCGTATGTGAGGAATGCCCAGACGGAACCT
ACTCCGATGAAGCCAACCACGTCGATCCGTGCCTGCCGTGCACCGTTTGCGA
GGACACAGAAAGGCAGCTGCGAGAGTGCACTCGGTGGGCCGACGCCGAATG
CGAAGAAATCCCTGGACGCTGGATCACCAGGTCTACTCCCCCTGAAGGGAGT
GACTCAACTGCACCTTCAACCCAGGAGCCTGAAGCTCCCCCAGAGCAGGACC
TCATCGCTTCAACAGTGGCCGGGGTGGTCACTACGGTGATGGGGAGTTCTCA
GCCTGTGGTCACACGAGGCACCACAGATAACCTCATTCCGGTTTACTGCTCA
ATCTTGGCGGCAGTTGTGGTTGGTCTGGTGGCGTATATCGCGTTTAAAAGAT
GGAATAGCTAGTAGGCGGCCGC

Legend:

5’ region

Leader sequence and Kozak sequence

Anti-CD20 scFv

CD8α-hinge,TM

4-1BBcyto

CD3ζcyto

T2A

LNGFR

3’ region

Anti-HER2-41BBζ (entire coding region inserted into MSGV1 vector, AgeI and NotI sites)

GGTACCTCACCCTTACCGAGTCGGCGACACAGTGTGGGTCCGCCGACACCAG
ACTAAGAACCTAGAACCTCGCTGGAAAGGACCTTACACAGTCCTGCTGACCA
CCCCCACCGCCCTCAAAGTAGACGGCATCGCAGCTTGGATACACGCCGCCCA
CGTGAAGGCTGCCGACCCCACGCGTCAAGTGGAGCAAGGCAGGTGGAAGCT
TCACCATGGAGACAGACACACTCCTGCTATGGGTGCTGCTGCTCTGGGTTCC
AGGTTCCACAGGTGATATACAAATGACCCAATCTCCCTCCTCATTGAGTGCCT
CAGTCGGGGATCGCGTAACAATCACTTGCAAAGCTAGTCAAGATGTATCTAT
TGGTGTGGCATGGTACCAGCAGAAACCAGGGAAAGCGCCTAAACTCCTTATC
TATAGCGCGAGCTATCGATATACAGGAGTGCCGTCCAGATTTAGCGGTTCCG
GTTCAGGAACAGACTTTACGCTGACCATATCTTCACTTCAACCGGAAGATTTC
GCGACTTACTACTGCCAACAATATTATATCTACCCTTATACTTTCGGCCAAGG
GACTAAAGTTGAAATAAAAGGGGGTGGCGGGTCTGGCGGTGGAGGATCTGG
CGGCGGTGGTTCTGAAGTCCAATTGGTTGAAAGTGGTGGTGGGTTGGTCCAG
CCTGGCGGATCCTTGCGGTTGTCCTGTGCCGCCTCAGGATTTACCTTCACAG
ATTACACGATGGACTGGGTGCGACAGGCCCCAGGTAAGGGCCTTGAATGGG
TAGCTGACGTAAACCCTAACAGCGGGGGCTCCATATACAATCAACGGTTCAA
AGGACGGTTTACACTGTCTGTCGACAGGAGTAAGAATACCCTCTATCTCCAG
ATGAACTCTCTCCGAGCTGAGGACACGGCAGTATATTACTGTGCTCGAAACC
TCGGACCCTCTTTCTATTTCGACTACTGGGGTCAGGGCACGCTGGTGACTGT
GAGCAGTACCACTACTCCGGCACCGCGCCCCCCAACTCCTGCACCGACGATA
GCTTCACAACCGCTTTCATTGCGGCCCGAAGCATGTCGGCCAGCCGCCGGAG
GCGCTGTGCATACAAGAGGGCTGGATTTTGCATGTGATATATATATTTGGGC

GCCCCTTGCTGGCACTTGCGGCGTTCTTCTTCTTAGCCTCGTTATTACGCTCT
ACTGTAAGCGAGGTAGGAAAAAATTGCTGTATATCTTTAAACAGCCTTTTATG
AGACCCGTGCAAACGACTCAAGAGGAAGACGGGTGTAGCTGTAGATTTCCTG
AAGAGGAAGAGGGGGGGTGCGAACTGCGGGTGAAGTTCAGCAGAAGCGCCG
ACGCCCCTGCCTACCAGCAGGGCCAGAATCAGCTGTACAACGAGCTGAACCT
GGGCAGAAGGGAAGAGTACGACGTCCTGGATAAGCGGAGAGGCCGGGACCC
TGAGATGGGCGGCAAGCCTCGGCGGAAGAACCCCCAGGAAGGCCTGTATAA
CGAACTGCAGAAAGACAAGATGGCCGAGGCCTACAGCGAGATCGGCATGAA
GGGCGAGCGGAGGCGGGGCAAGGGCCACGACGGCCTGTATCAGGGCCTGTC
CACCGCCACCAAGGATACCTACGACGCCCTGCACATGCAGGCCCTGCCCCCA
AGGCTCGAGTAATAAGCGGCCGC

Legend:

5’ region

Anti-HER2 CAR

3’ region

Table S5 | Fitted parameter values.

SNAP-CAR T cell parameters					
Antibody	***k_f1***	***k_f2***	***k_r2***	***[Tcell]***	***[Tumor Cells]***
FMC63	3.91606E-05	0.000113643	0.002273569	0.269805904	0.77409019
Cetuximab	0.000028	0.0031	0.0058	0.166053904	2.435457259
Herceptin	2.35329E-05	5.2682E-05	4.6763E-05	0.023368542	3.662540407
Rituximab	5.52577E-06	5.55581E-05	0.001082705	0.204688756	6.649347881
synNotch T cell parameters					
Antibody	***k_f1***	***k_f2***	***k_r2***	***[Tcell]***	***[Tumor Cells]***
FMC63	1.97257E-05	0.000588937	0.000973278	0.350870732	2.074613689
Cetuximab	6.88057E-06	0.002739548	0.004410671	0.153193991	7.998250675
Herceptin	7.97625E-06	0.00016536	1.03176E-05	0.89801904	15.61597465
Rituximab	2.80993E-06	0.000111117	0.000665739	1.549807386	7.599804046
