# Supplementary material for: Post-translational covalent assembly of CAR and synNotch receptors for programmable antigen targeting
Source: Nat Commun. 2023 May 9;14:2463. doi: 10.1038/s41467-023-37863-5 (PMC10169838; doi:10.1038/s41467-023-37863-5)
Supplement: Supplementary file 2 — Reporting Summary [file 41467_2023_37863_MOESM2_ESM.pdf]

nature portfolio

Corresponding author(s): Jason Lohmueller
Last updated by author(s): Apr 2, 2023

Reporting Summary

Nature Portfolio wishes to improve the reproducibility of the work that we publish. This form provides structure for consistency and transparency in reporting. For further information on Nature Portfolio policies, see our Editorial Policies and the Editorial Policy Checklist.

Statistics

For all statistical analyses, confirm that the following items are present in the figure legend, table legend, main text, or Methods section.

n/a	Confirmed	
☐	☒	The exact sample size (*n*) for each experimental group/condition, given as a discrete number and unit of measurement
☐	☒	A statement on whether measurements were taken from distinct samples or whether the same sample was measured repeatedly
☐	☒	The statistical test(s) used AND whether they are one- or two-sided<br>*Only common tests should be described solely by name; describe more complex techniques in the Methods section.*
☒	☐	A description of all covariates tested
☐	☒	A description of any assumptions or corrections, such as tests of normality and adjustment for multiple comparisons
☐	☒	A full description of the statistical parameters including central tendency (e.g. means) or other basic estimates (e.g. regression coefficient) AND variation (e.g. standard deviation) or associated estimates of uncertainty (e.g. confidence intervals)
☐	☒	For null hypothesis testing, the test statistic (e.g. *F*, *t*, *r*) with confidence intervals, effect sizes, degrees of freedom and *P* value noted<br>*Give P values as exact values whenever suitable.*
☒	☐	For Bayesian analysis, information on the choice of priors and Markov chain Monte Carlo settings
☒	☐	For hierarchical and complex designs, identification of the appropriate level for tests and full reporting of outcomes
☒	☐	Estimates of effect sizes (e.g. Cohen's *d*, Pearson's *r*), indicating how they were calculated

Our web collection on statistics for biologists contains articles on many of the points above.

Software and code

Policy information about availability of computer code

Data collection	Software used it stated in the manuscript (BD FACSDiva (v9.0), Living Image v4.3.1, Softmax Pro 7, Image Lab Version 6.1.0).
Data analysis	Flow cytometery data was analyzed using FlowJo v10.8.1 (FlowJo, LLC) and data for all figures was presented and analyzed using Graphpad Prism v9. (GraphPad Software, LLC). The ODE model was written in Python3.7 and solved using SciPy1.7.3. Code is publicly available: https://github.com/pitt-miskov-zivanov-lab/TernaryBody

For manuscripts utilizing custom algorithms or software that are central to the research but not yet described in published literature, software must be made available to editors and reviewers. We strongly encourage code deposition in a community repository (e.g. GitHub). See the Nature Portfolio guidelines for submitting code & software for further information.

Data

Policy information about availability of data

All manuscripts must include a data availability statement. This statement should provide the following information, where applicable:

- Accession codes, unique identifiers, or web links for publicly available datasets
- A description of any restrictions on data availability
- For clinical datasets or third party data, please ensure that the statement adheres to our policy

All data generated and analyzed during this study are included in this published article and its Supplementary Information. Source data are provided with this paper.

Human research participants

Policy information about studies involving human research participants and Sex and Gender in Research.

Reporting on sex and gender	N/A
Population characteristics	N/A
Recruitment	N/A
Ethics oversight	N/A

Note that full information on the approval of the study protocol must also be provided in the manuscript.

Field-specific reporting

Please select the one below that is the best fit for your research. If you are not sure, read the appropriate sections before making your selection.

☒ Life sciences ☐ Behavioural & social sciences ☐ Ecological, evolutionary & environmental sciences

For a reference copy of the document with all sections, see nature.com/documents/nr-reporting-summary-flat.pdf

Life sciences study design

All studies must disclose on these points even when the disclosure is negative.

Sample size	Statistical methods were not used to predetermine sample size. Sample sizes for experiments were determined from analogous studies performed by us and others where the differences between groups were expected to be comparable, and the same statistical methods could be applied. A minimum of triplicates was chosen to allow for calculation of statistics. For flow cytometry, >10,000 events were collected to characterize a distribution of the data. Biologically independent experimental triplicates then allowed for statistical analysis of data features.
Data exclusions	No data were excluded.
Replication	All key experiments were performed at least twice. Results were consistent across these replicates and the data presented in the article is representative of the trends we observed.
Randomization	Samples were allocated to identical wells in a spatially in a manner that facilitated experimental organization. There is no reason to believe the spatial location of the sample influenced experimental results. For in vivo mouse experiments, after tumor injection, but before experimental treatment, mice were evaluated for tumor growth and evenly distributed for luminescence with each group having some larger and some smaller tumor sizes. This re-distribution was performed to ensure that post-therapy tumor growth was not affected by the tumor size prior to therapeutic treatment as larger initial tumors could be expected to grow more rapidly.
Blinding	Blinding was not performed, however, the authors agree that samples were processed uniformly when acquiring data regardless of whether they were controls or experimental samples. As the data presented is quantitative in nature, blinding was unnecessary for the experiments performed.

Reporting for specific materials, systems and methods

We require information from authors about some types of materials, experimental systems and methods used in many studies. Here, indicate whether each material, system or method listed is relevant to your study. If you are not sure if a list item applies to your research, read the appropriate section before selecting a response.

Materials & experimental systems

n/a	Involved in the study	
☐	☒	Antibodies
☐	☒	Eukaryotic cell lines
☒	☐	Palaeontology and archaeology
☐	☒	Animals and other organisms
☒	☐	Clinical data
☒	☐	Dual use research of concern

Methods

n/a	Involved in the study	
☒	☐	ChIP-seq
☐	☒	Flow cytometry
☒	☐	MRI-based neuroimaging

Antibodies

Antibodies used	CD69-PE (BD Biosciences, CAT#555531), 1:100; CD69-BV711(BioLegend, CAT#310944), 1:100; CD62L-FITC(BD Biosciences, CAT#555543), 1:100; CD25-APC,(BD Biosciences, CAT#555434), 1:100; CD107a-APC(BD Biosciences, CAT#560664), 1:100; CD271 (LNGFR)-BV421(BD Biosciences, CAT#562562), 1:100; CD4-BUV395(BD Biosciences, CAT#563550), 1:100; CD8-PE-Cy7(BioLegend, CAT#344750), 1:100; humanIgGFcgamma-AF647(Jackson ImmunoResearch, CAT#709-606-098), 1:200; humanIgG(H+L)-AF647 (Jackson ImmunoResearch, CAT#109-605-003), 1:200; mycTag-AF488(Cell Signaling Technology, CAT#2279S), 1:50; CD19-PE (BioLegend, CAT#363003), 1:100; CD19-BV605(BD Biosciences, CAT#562653), 1:100; CD20-BV421(BioLegend, CAT#302330), 1:100; CD45RA-BV785(BioLegend, CAT#304140), 1:100; CD3-BUV496(BD Bioscience, CAT#564809), 1:100; CD271-PE(BD Biosciences, CAT#557196), 1:100;
Validation	Validation provided by supplier e.g., antibodies from BioLegend and BD Bioscience have been widely used and their use is cited on their websites. Additionally, each antibody was tested on cells known to be negative and positive for the targeted antigen e.g. the anti-CD271(LNGFR) antibody was tested on un-transduced (MOCK) primary human T cells and CAR-transduced primary human T cell populations.

Eukaryotic cell lines

Policy information about cell lines and Sex and Gender in Research

Cell line source(s)	Jurkat Clone E6-1 (TIB-152), ZR-75-1(CRL-1500), K562 (CCL-243), SKOV-3(HTB-77), HEK293T cells (ATTC, CRL-3216), and Raji (CCL-86) were obtained from American Type Culture Collection (ATCC). NALM6 cells stably expressing firefly luciferase were a gift from G. Delgoffe (University of Pittsburgh), for which parental NALM-6 was originally sourced from ATCC (CRL-3273). HEK293-GP cells were a gift from U. Kammula (University of Pittsburgh) for which parental HEK293 cells originally sourced from ATCC (CRL 1573). K562+EGFRt, K562+CD20, NALM6+CD20, NALM6+HER2, and Jurkat+EGFRt cells that stably express full-length antigens or EGFRt, were generated by transducing cells with the indicated tumor antigen expressing lentivirus and sorting for cells positive for antigen expression. To create the SNAP-CAR stable cell line, Jurkat cells were transduced with SNAP-41BBζ, and underwent fluorescence-activated cell sorting (FACS) for TagBFP expression and reporter (mCherry+) expression. To generate SNAP-synNotch lines, SNAP-synNotch-Gal4VP64 was co-transduced with either pHR-Gal4UAS-tBFP-PGKmCherry or pHR-Gal4UAS-IL7-PGKmCherry lentivirus, and receptor and response construct positive cells were obtained by FACS for anti-Myc-Tag antibody staining (Cell signaling Technology) and mCherry expression, respectively.
Authentication	Cell lines were authenticated for for antigen expression by flow cytometry staining using indicated antibodies as shown in Supplmentary Figure S2.
Mycoplasma contamination	Cells were routinely tested for mycoplasma contamination, and all of the lines used tested negative for mycoplasma.
Commonly misidentified lines (See ICLAC register)	From the list no lines except HEK293T cells were used. HEK293T cells were used for lentivirus production due to the ease of transfecting them with viral plasmids.

Animals and other research organisms

Policy information about studies involving animals; ARRIVE guidelines recommended for reporting animal research, and Sex and Gender in Research

Laboratory animals	mice NOD-SCID-ɣchain-deficient (NSG) (JAX mice), 4-6weeks of age
Wild animals	None.
Reporting on sex	Only female mice were used in the study for relevance to ovarian and breast cancers with the HER2 antigen and to eliminate any potential issues with redistributing mice to balance tumor size post tumor injection (and before experimental treatment). All mice were purchased directly and sex was verified by the vendor (JAX mice). All mice were housed in specific pathogen free conditions at an ambient temperature of 20-26°C and humidity of 30-70% with a 12:12 hour light dark cycle.
Field-collected samples	None.
Ethics oversight	Animal work in this study was approved by the University of Pittsburgh Institutional Animal Care and Use Committee (IACUC), and procedures were performed under their guidelines.

Note that full information on the approval of the study protocol must also be provided in the manuscript.

Flow Cytometry

Plots

Confirm that:

- [x] The axis labels state the marker and fluorochrome used (e.g. CD4-FITC).
- [x] The axis scales are clearly visible. Include numbers along axes only for bottom left plot of group (a 'group' is an analysis of identical markers).
- [x] All plots are contour plots with outliers or pseudocolor plots.
- [x] A numerical value for number of cells or percentage (with statistics) is provided.

Methodology

Sample preparation	For analyzing cultured cells, cells were washed and resuspended in flow cytometry buffer (PBS + 2% FBS) and then stained using fluorescently antibodies for 30 minutes at 4°C followed by two washes and a final resuspension in flow cytometry buffer. For isolation and analysis of mouse blood, 100-150uL of blood was drawn into 500uL of Alsever's solution anti-coagulant and incubated for 20-30min at ambient temperature. 1mL of RBC lysis buffer was added and cells were spun at 500xg for 2 minutes. The pelleted cells then underwent RBC lysis (2mL of RBC lysis Buffer and incubated for 2min on ice) and two washes with FACS buffer. Cells were then stained with antibodies and washed similar to cultured cells above. For preparation of mouse splenocytes, spleens were extracted into RPMI+10%FBS, mashed and filtered through a 70µM filter, washed 2 times in FACS buffer, underwent RBC lysis, washing, and antibody staining as noted above.
Instrument	All experiments were run on the BD LSRFortessa flow cytometer.
Software	BD FACSDiva (v9.0) was used for data collection. Data was analyzed using FlowJo v10.8.1 (FlowJo, LLC), and statistical analysis and plotting were performed using Graphpad Prism v9. (GraphPad Software, LLC).
Cell population abundance	At least 10,000 relevant events were acquired for all flow cytometry analyses.
Gating strategy	First an FSC-Area/SSC-Area gate is drawn to remove debris and dead cells. Subsequent FSC-Area/FSC-Height SSC-Area/SSC-Height gates are then used to identify singlet events. Where relevant multi-color samples were compensated with the help of single-color controls. To define the boundaries between "+" and "-" gates are defined by identically treated true-negative samples or the same sample stained with an isotype control antibody.

- [x] Tick this box to confirm that a figure exemplifying the gating strategy is provided in the Supplementary Information.
